# Unlocking the potential: unveiling tyrphostins with Michael-reactive cyanoacrylate motif as promising inhibitors of human 5-lipoxygenase

**DOI:** 10.1007/s00424-024-03019-7

**Published:** 2024-09-30

**Authors:** Maximilian Molitor, Amelie Menge, Sebastian Mandel, Sven George, Susanne Müller, Stefan Knapp, Bettina Hofmann, Dieter Steinhilber, Ann-Kathrin Häfner

**Affiliations:** 1 Institute of Pharmaceutical Chemistry, Goethe University, Max-von-Laue-Str. 9, 60438 Frankfurt Am Main, Germany; 2 Buchmann Institute for Molecular Life Sciences and Structural Genomics Consortium (SGC), Max-von-Laue-Str. 15, 60438 Frankfurt Am Main, Germany

**Keywords:** Inflammation, 5-Lipoxygenase, Michael acceptors, Leukotrienes, Tyrphostin

## Abstract

Human 5-lipoxygenase (5-LO) is the key enzyme in the biosynthesis of leukotrienes, mediators of the innate immune system that also play an important role in inflammatory diseases and cancer. In this study, we present compounds, containing a Michael-reactive cyanoacrylate moiety as potent inhibitors of 5-LO. Representatives of the tyrosine kinase inhibitor family called tyrphostins, structurally related to known 5-LO inhibitors, were screened for their 5-LO inhibitory properties using recombinant human 5-LO, intact human PMNL (polymorphonuclear leukocytes), and PMNL homogenates. Their mode of action was characterized by the addition of glutathione, using a fourfold cysteine 5-LO mutant and mass spectrometry analysis. SAR studies revealed several members of the tyrphostin family containing a Michael-reactive cyanoacrylate to efficiently inhibit 5-LO. We identified degrasyn (IC_50_ 0.11 µM), tyrphostin A9 (IC_50_ 0.8 µM), AG879 (IC_50_ 78 nM), and AG556 (IC_50_ 64 nM) as potent 5-LO inhibitors. Mass spectrometry analysis revealed that degrasyn and AG556 covalently bound to up to four cysteines, including C416 and/or C418 which surround the substrate entry site. Furthermore, the 5-LO inhibitory effect of degrasyn was remarkably impaired by the addition of glutathione or by the mutation of cysteines to serines at the surface of 5-LO. We successfully identified several tyrphostins as potent inhibitors of human 5-LO. Degrasyn and AG556 were able to covalently bind to 5-LO via their cyanoacrylate moiety. This provides a promising mechanism for targeting 5-LO by Michael acceptors, leading to new therapeutic opportunities in the field of inflammation and cancer.

## Introduction

Covalent inhibitors have gained significant interest as drug candidates in the last decades. Despite previous concerns regarding toxicity and off-target effects, they offer several advantages, including enhanced selectivity, prolonged effect or residence time, lower risk of drug resistance, and high potency [45, 50]. This progress has led to a number of approvals of Michael acceptor containing drugs, particularly kinase inhibitors such as afatinib, osimertinib, or zanubrutinib, but also for other target classes such as the recently approved KRAS inhibitor sotorasib [1, 16, 37, 51]. All of these compounds covalently bind to nucleophilic amino acids due to the presence of a Michael acceptor. The amino acids targeted by currently used electrophilic moieties, the so-called warhead, are cysteine, lysine, or serine/threonine [5]. This study focuses specifically on inhibitors of human 5-lipoxygenase (5-LO) that contain a cyanoacrylate warhead covalently targeting cysteine residues.

Human 5-lipoxygenase (5-LO) encoded by the gene ALOX5 has been the focus of the development of anti-inflammatory drugs for many years. It is the key enzyme of leukotriene (LT) biosynthesis catalyzing the conversion of arachidonic acid (AA) to leukotriene A_4_ (LTA_4_) in a two-step reaction [43]. LTs are potent pro-inflammatory mediators that act as phagocyte chemoattractants or smooth muscle contractors [11, 39]. Therefore, they play a major role in the innate immune response, as well as in pathophysiological conditions such as asthma, allergic inflammation, and cardiovascular diseases [39]. Novel 5-LO inhibitors may have tremendous potential in the management of LT-dependent diseases.

Apart from the iron-ligand zileuton, the only FDA-approved 5-LO inhibitor that suffers from potentially hepatotoxic effects and an unfavorable pharmacokinetic profile, a multitude of other 5-LO inhibitory structures have been discovered [8, 49, 55]. Some of the identified inhibitors, such as U73122, act as Michael acceptors that can covalently bind to nucleophiles such as cysteines [19, 35]. U73122 was one of the first Michael-reactive 5-LO inhibitors described, having a highly reactive maleimide group. It has been demonstrated that the mode of action of U73122 involves binding to cysteines C416 and C418 in 5-LO [19]. Unselective reactions with cysteines and irreversible binding can be detrimental to drug development. In the case of U73122, a major loss of potency has been reported in the presence of glutathione (GSH), which is present at high concentration in the cytoplasm of cells [12]. Furthermore, the 5-LO inhibitor AA-861 and thymoquinone act as Michael acceptors via their benzoquinone moiety [35, 58]. Nitro fatty acids such as nitro-oleic acid represent another class of Michael acceptor that inhibits 5-LO via cysteine C416 and C418 binding [2, 18]. Here, we investigated cyanoacrylates and cyanoacrylamides as Michael-reactive moieties as they can be easily incorporated into known 5-LO inhibitors such as caffeic acid phenethyl ester (CAPE). Additionally, they exhibit reversible binding with adjustable reactivity [41, 47]. Reversible covalent binding offers advantages as it enhances the residence time at the target but also leads to reduced off-target toxicity [3, 7]. Inspired by CAPE, we used 2-cyanocinnamic acid (CCA) as a lead structure for the search for potent novel 5-LO inhibitors. This structure is a key element of many members of the tyrphostins, a well-known group of tyrosine kinase inhibitors [31]. First described in the 1980s as epidermal growth factor receptor (EGFR) inhibitors, they represent early advances in kinase inhibitor development [56]. Therefore, we selected commercially available compounds that either had a CCA motif or similarities to CAPE or contained a benzylidenemalononitrile (BMN) structure [13, 14, 38]. These compounds were analyzed for their 5-LO inhibition and selectivity, and the covalent binding mode of selected compounds was investigated. This approach successfully identified a significant number of small molecules with 5-LO inhibitory activity, such as degrasyn, tyrphostin A9, AG879, and AG556, which stood out due to their selectivity for 5-LO compared to other LOs and exceptional potency. It is noteworthy that degrasyn and AG556 demonstrated covalent binding to cysteines close to the substrate entry site of 5-LO, making them a novel type of covalent 5-LO inhibitors containing cyanoacrylate motifs.

## Materials and methods

### Materials

Calcium ionophore A23187 and benzylidene malononitrile (BMN) were provided by Sigma Aldrich (Darmstadt, Germany). AA, CAPE, prostaglandin B_1_ (PGB_1_), AG82, AG99, AG126, AG494, and AG556 were purchased from Cayman Chemical (Ann Arbor, MI, USA). Tyrphostin A1, tyrphostin A9, AG18, AG30, (*E*)-AG99, AG528, AG825, AG879, AG1024, degrasyn, RG13022, RG14620, SU1498, and WP1066 were obtained from MedChemExpress LLC (Monmouth Junction, NJ, USA). Enamine Ltd. (Kyiv, Ukraine) provided CCA and tyrphostin 8. ATP, tris(2-carboxyethyl)phosphine (TCEP), GSH, and the UHPLC solvents acetonitrile, methanol, and acetic acid were purchased from Carl Roth GmbH (Karlsruhe, Germany). Lymphocyte separation medium 1077 was provided by PromoCell, Heidelberg, Germany. Dulbecco’s Modified Eagle Medium (DMEM), fetal bovine serum (FBS), L-glutamine penicillin/streptomycin, PBS (all Gibco™) for cell culture and Hoechst33342, MitoTracker red, and annexin V Alexa Fluor 680 conjugate were purchased from Fisher Scientific (Waltham, MA, USA). 384 well µClear® f-bottom plates for cell culture were purchased from Greiner (Kremsmünster, Austria), BioTracker™ 488 Green Microtubule Cytoskeleton Dye from EMD Millipore (Burlington, MA, USA).

### Isolation and purification of recombinant 5-LO

Recombinant 5-lipoxygenase wild type (r5-LO_WT) and its cysteine mutant (r5-LO_4C), where the cysteines 159, 300, 416, and 418 were exchanged to serines, were expressed in BL21 (DE3) *E. coli* and purified using an ÄKTAxpress system (GE Healthcare, Uppsala, Sweden) as published before [17, 19, 46]. The elution fractions were checked by SDS-PAGE and Coomassie staining and further purified by size exclusion chromatography using an ÄKTA Purifier 10 with a HiLoad 16/60 Superdex 200 column (Cytiva, Marlborough, MA, USA) in gel filtration buffer (20 mM HEPES pH 7.4, 150 mM NaCl, 1 mM EDTA, 0.5 mM ATP, 0.5 mM TCEP, and 5% (v/v) glycerol). The final elution fractions were controlled for impurities by SDS-PAGE and Coomassie staining before the protein was concentrated to 2–4 mg/mL using Amicon® ultra centrifugal filters (Merck Millipore, Darmstadt, Germany) and snap frozen in liquid nitrogen. Protein concentration was determined with a Bradford protein assay (Bio-Rad, Hercules, CA, USA). The protein was stored at − 80 °C and thawed quickly for immediate use.

### Isolation of human PMNL and platelets

For the isolation of polymorphonuclear leukocytes (PMNL) and platelets, human leukocyte concentrates were purchased from DRK-Blutspendedienst (Frankfurt, Germany). Donors gave written consent for use in research. PMNL were isolated as described previously [27]. In brief, after dextran sedimentation, the supernatant was used for gradient centrifugation by gradually overlaying lymphocyte separation medium 1077 with supernatant. The sample was centrifuged for 10 min at 1700 × g. For platelet isolation, 25 mL of the upper layer of the resulting gradient was taken, mixed with 20 mL PBS pH 5.9, and centrifuged at 1850 × g for 10 min at RT. The pellet was resuspended in a 1:1 mix of PBS pH 5.9 and 0.9% (m/m) NaCl solution. The cells were centrifuged at 1850 × g for 10 min and resuspended in PBS pH 5.9 for further use. In order to isolate PMNL, the pellet after density centrifugation underwent a hypotonic lysis of excess erythrocytes and the cells were resuspended in PBS pH 7.4 containing 1 mg/mL glucose for subsequent use.

### Determination of r5-LO activity

Three micrograms of r5-LO_WT, r5-LO_4C, or a r5-LO single cysteine mutant (C159S, C300S, C416S, or C418S) was preincubated with inhibitor in 1 mL PBS containing 1 mM EDTA and 1 mM ATP (PBS/EDTA/ATP) for 15 min on ice. In experiments involving GSH, the inhibitors were preincubated in PBS/EDTA/ATP containing 1 mM GSH. After warming the sample for 20 s at 37 °C, the reaction was started by the addition of 2 mM CaCl_2_ and 10 µM AA. Each sample was incubated further for 10 min at 37 °C until the reaction was stopped by the addition of 1 mL ice-cold methanol. For further analysis, the samples were subjected to solid phase extraction and analyzed via UHPLC with UV/MS detection using a H-class Acquity UPLC (Waters, Milford, MA, USA) as described previously [26]. The reaction products of 3 µg of protein were quantified using PGB_1_ as internal standard and include 5-hydro(per)oxyeicosatetraenoic acid (H(p)ETE), 5-oxo-ETE, 6-*trans*-12-*epi*-LTB_4_, 6-*trans*-LTB_4_, and LTB_4_. The results were normalized to DMSO controls. The final DMSO concentration in all samples was 1%. Each compound was tested three to six times up to a concentration of 30 µM, and IC_50_ values (with 95% CI) were calculated. Then, 0.3 µM of BWA4C was used as a positive control for 5-LO inhibition.

### Determination of 5-LO, 12-LO, and 15-LO activity in intact cells

5 × 10^6^ PMNL or 1 × 10^8^ platelets were resuspended in 1 mL PBS containing 1 mg/mL glucose and 1 mM CaCl_2_ (PGC). All compounds investigated were preincubated with the cell suspension for 15 min at 37 °C before starting the reaction by the addition of 20 µM AA and 2.5 µM calcium ionophore A23187. After 10 min, the reaction was stopped by adding 1 mL ice-cold methanol. Further sample processing was performed as stated before. 12-LO and 15-LO product formation was detected as 12-HETE and 15-HETE, respectively. All experiments were repeated three to six times with concentrations up to 30 µM, and the results were normalized on 1% DMSO control.

### Determination of 5-LO activity in cell homogenates

Cell homogenates were prepared by resuspending 7.5 × 10^6^ PMNL in 1 mL PBS with 1 mM EDTA and sonicating for 3 × 10 s. Samples were kept on ice, and 1 mM ATP or 1 mM ATP/1 mM GSH was added. The inhibitors were preincubated with the cell homogenate on ice for 15 min before heating the samples at 37 °C for 30 s. The reaction was started by the addition of 20 µM AA and 2 mM CaCl_2_ and stopped after 10 min by the addition of 1 mL ice-cold methanol. Solid phase extraction and UPLC analysis were performed as described above. All experiments were repeated three times with inhibitor concentrations up to 30 µM, and the results were normalized on 1% DMSO control.

### Preparation of samples for mass spectrometry

r5-LO_WT or r5-LO_4C protein was diluted to 20 µM in gel filtration buffer; 100 µM of compound was added and incubated for a minimum time of 1 h at RT before 10 µL was injected onto a 1260 Infinity HPLC (Agilent, Santa Clara, USA) using a C3 column with a flow rate of 0.4 mL/min as published before [44]. The HPLC was coupled with a 6230 electrospray ionization TOF LC/MS detector (Agilent, Santa Clara, CA, USA). Data were acquired using the Agilent MassHunter Acquisition software and processed with the Agilent Mass Hunter BioConfirm software (B.08.00). Peak intensities were calculated and normalized to the highest count of each data set with GraphPad Prism version 7.05 (GraphPad Software, Boston, MA, USA).

### Multiplex assay

For the assessment of cell health, a live-cell high-content screen was performed in U2OS (ATCC®HTB-96™) osteosarcoma cells as described previously [52, 53]. In brief, cells were cultured in DMEM plus L-glutamine (High glucose) supplemented by 10% FBS and penicillin/streptomycin. Cells were seeded at a density of 1250 cells per well in a 384-well plate in culture medium, with a volume of 50 µL per well. All outer wells were filled with 100 µL PBS. Simultaneously with seeding, cells were stained with 60 nM Hoechst33342, 75 nM MitoTracker red, 0.3 µL/well annexin V Alexa Fluor 680 conjugate, and 25 nL/well BioTracker™ 488 Green Microtuble Cytoskeleton dye. Cell shape and fluorescence were measured before and 12, 24, and 48 h after compound treatment using a CQ1 high-content confocal microscope (Yokogawa, Musashino, Japan). The following setup parameters were used for image acquisition: Ex 405 nm/Em 447/60 nm, 500 ms, 50%; Ex 561 nm/Em 617/73 nm, 100 ms, 40%; Ex 488/Em 525/50 nm, 50 ms, 40%; Ex 640 nm/Em 685/40, 50 ms, 20%; bright field, 300 ms, 100% transmission, one centered field per well, and 7 z stacks per well with 55 µm spacing. The compounds were added in a 1:1000 dilution (50 nL/well) using an Echo 550 automated handling system (LabCyte, San Josef, California, USA) to a final concentration of 1 µM and 10 µM. Acquired images of the cells were processed using the Yokogawa CellPathfinder software (v3.04.02.02). Cells were detected and gated with a machine learning algorithm as described previously [53]. Results were normalized to cells exposed to 0.1% DMSO. All compounds were tested in biological duplicates with technical duplicates, and SD (standard deviation) was calculated for biological duplicates.

### Statistics

If not stated otherwise, results are depicted as mean ± standard deviation. For determining significance, a one-way ANOVA and if stated a multiple comparison *t*-test with Dunnett’s correction (95% confidence interval (CI)) was performed using GraphPad Prism version 7.05 (GraphPad Software, Boston, MA, USA). To calculate IC_50_ values, a non-linear regression using the least squares method with variable slope was chosen.

## Results

### Inhibition of 5-LO product formation by 2-cyanocinnamic acid and caffeic acid derivatives

Some years ago, a derivative of caffeic acid called caffeic acid phenethyl ester (CAPE) and its corresponding amide have been described as inhibitors of 5-LO-mediated LT biosynthesis in PMNL [6, 24]. When a nitrile group is introduced at the C2 position of caffeic acid, a cyanoacrylate structure is formed that may react as a reversible Michael acceptor, resulting in the structure of the known tyrosine kinase inhibitor tyrphostin AG30 [14]. In general, tyrphostins are a group of structurally diverse tyrosine kinase inhibitors with a large set of 2-cyanocynamic acid (CCA) derivatives [14, 31]. Therefore, we decided to screen a set of 22 commercially available compounds featuring these scaffolds for the inhibition of LT biosynthesis, starting with purified, recombinant 5-LO protein (r5-LO_WT) as well as intact PMNL. First, we analyzed derivatives of CCA (Table 1). While CCA itself and also AG30 which contains the cyanoacrylate motif showed only marginal 5-LO inhibiting activity, the exchange of the carboxylic acid of AG30 to the corresponding amide AG99 yielded an IC_50_ of 1.5 µM using the recombinant enzyme and an IC_50_ of 7.2 µM in intact PMNL. The derivatives AG825 and AG879 possess additional bulky residues; AG825 carries a benzothiazole and AG879 two isobutyl moieties. AG825 failed to inhibit 5-LO at concentrations up to 10 µM, whereas AG879 proved to be a potent inhibitor with IC_50_ values of 0.08 µM and 0.4 µM, respectively. In AG879, the amide is replaced by a thioamide and the phenyl ring has been modified to the di-*tert*-butylphenol motif, which is already known to inhibit 5-LO [36]. AG879 was first characterized as a Her2/ErbB2 inhibitor with an IC_50_ of 1 µM, which means that it inhibits 5-LO as effectively as its original target [30]. Next, we tested RG13022 and RG14620 that are phenylmethylene pyridine acetonitrile derivatives and lack a carbonyl function in proximity to the acrylonitrile group (Table 1).

**Table 1:**
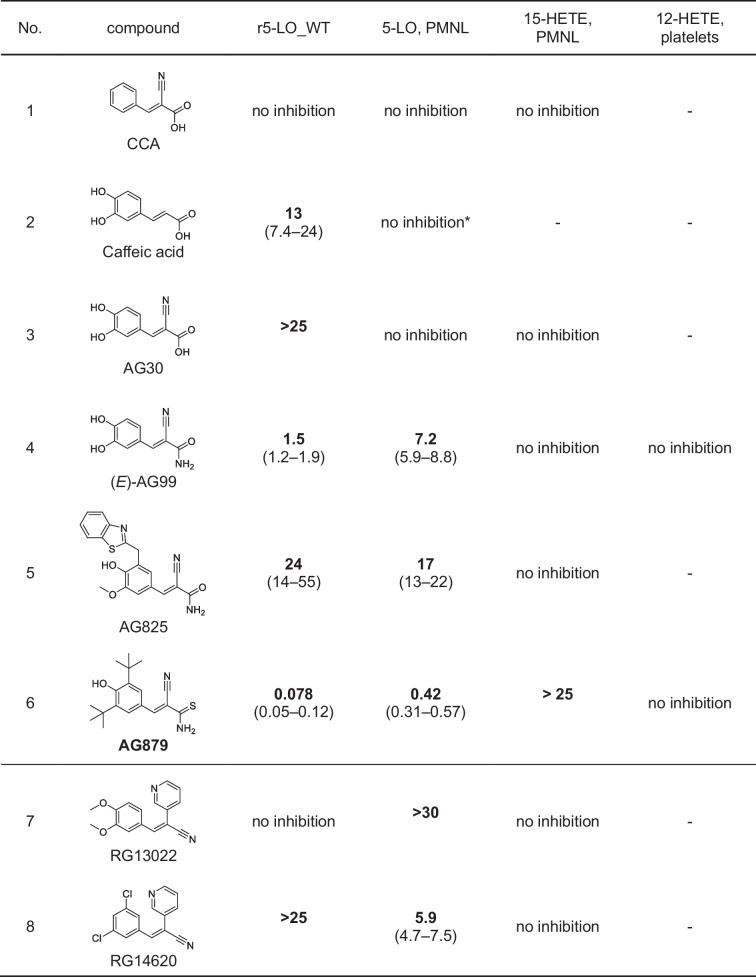
Inhibitory activities of test compounds on 5-LO product and 12- and 15-HETE formation. IC_50_ values (µM) (95% CI) for inhibition of LO product formation by test compounds with CCA scaffold using r5-LO_WT, intact PMNL, and platelets. *n* = 3–6, (-) not tested, *[35]

Both RG14620 and RG13022 are reported to be EGFR inhibitors in the micromolar range [48, 57]. Surprisingly, both compounds failed to inhibit r5-LO_WT or only showed marginal inhibitory activity, but we observed an inhibition of 5-LO product formation in PMNL by RG14620 with an IC_50_ of 5.9 µM.

Subsequently, we screened cyanoacrylates derived from CAPE, namely, AG494, AG490 (tyrphostin B42), AG556, AG528, SU1498, degrasyn (WP1130), and WP1066 (Table 2). In addition to containing a cyanoacrylate group, all compounds were structurally different from CAPE, being amides rather than esters. It should be noted that degrasyn and WP1066 stand out within this group as they lack phenolic hydroxyls and are instead brominated. All inhibitors of this class of compounds exhibited strong inhibition of r5-LO_WT in the nanomolar concentration range, with AG556 being the most potent inhibitor with an IC_50_ of 0.064 µM followed by AG528 (0.10 µM), degrasyn (0.11 µM), AG490 (0.28 µM), AG494 (0.36 µM), SU1498 (0.45 µM), and WP1066 (0.94 µM). In intact PMNL, all inhibitors also showed a good effect on 5-LO product formation.

**Table 2:**
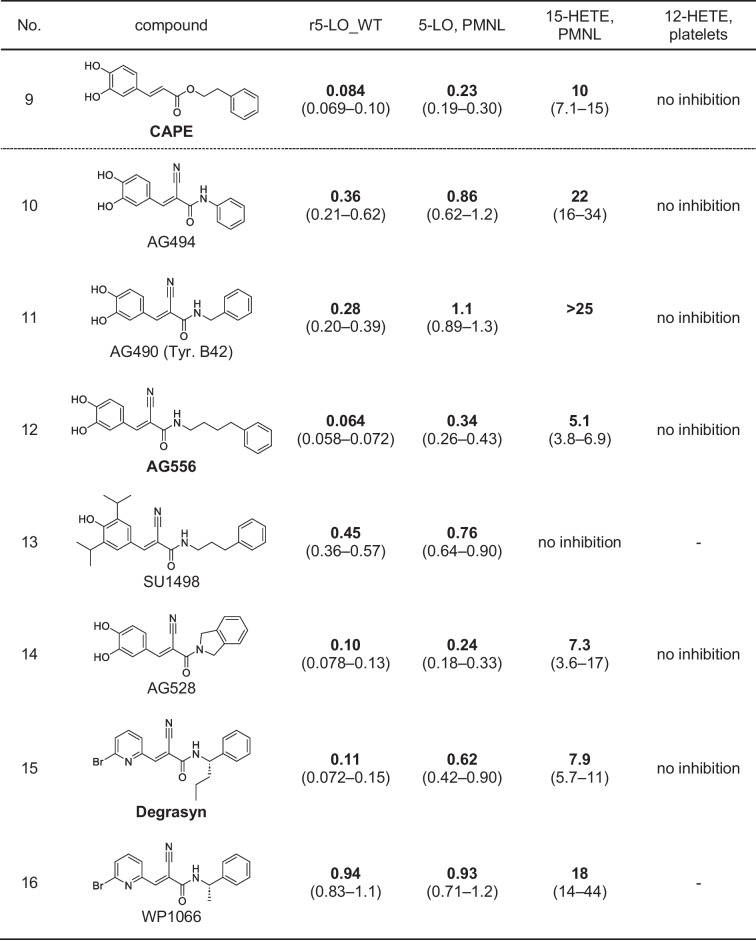
Inhibitory activities of test compounds on 5-LO product and 12- and 15-HETE formation. IC_50_ values (µM) (95% CI) for inhibition of LO product formation by tested CAPE-like compounds using r5-LO_WT, intact PMNL, and platelets. *n* = 3–6, (-) not tested

Last, we screened several tyrphostins that are derivatives of benzylidenemalononitrile (BMN) (Table 3) [14]. BMN as well as tyrphostin A1 and tyrphostin 8 failed to inhibit LT biosynthesis. Interestingly, as soon as a second hydroxyl group was added and thus a catechol motif was present, the resulting compound AG18 inhibited r5-LO_WT and LT biosynthesis in PMNL in the low micromolar concentration range. AG82 contains a third hydroxyl group at the benzene ring; however, this modification did not lead to further potency improvement. Both compounds had IC_50_ values of about 1 µM on r5-LO_WT, whereas in intact PMNL, the IC_50_ values of AG18 and AG82 increased to 5.4 µM and 8.0 µM, respectively. Interestingly, in AG126 where the hydroxyl group in meta-position is replaced by a nitro group, 5-LO inhibition was dramatically decreased. However, in line with the strong inhibitory effect of AG879, the identical di-*tert*-butylphenol substitution pattern also led to potent 5-LO inhibition by a BMN scaffold (tyrphostin A9), resulting in IC_50_ values of 0.80 µM (r5-LO_WT) and 1.1 µM (PMNL). Interestingly, the replacement of one of the isobutyl groups by a bromine atom as in AG1024 resulted in loss of activity for r5-LO_WT (~ 17 µM), while the inhibition of LT synthesis in PMNL was maintained (IC_50_ of 3.1 µM).

**Table 3:**
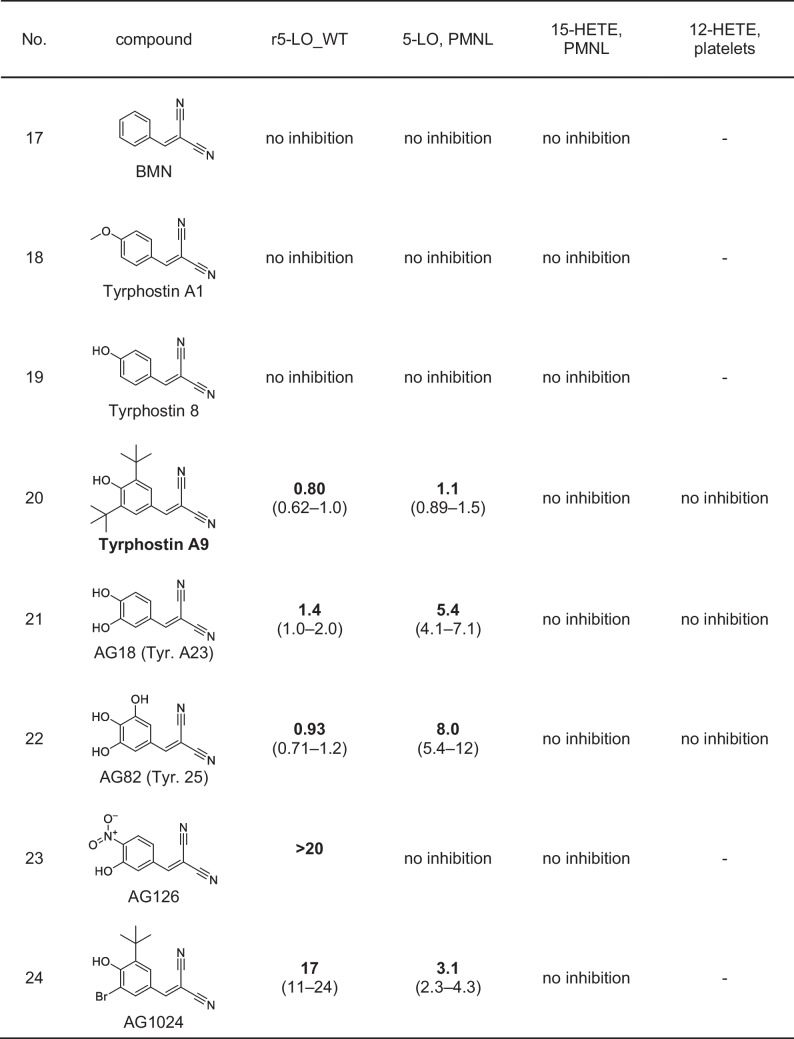
Inhibitory activities of test compounds on 5-LO product and 12- and 15-HETE formation. IC_50_ values (µM) (95% CI) for inhibition of LO product formation by tested BMNs using r5-LO_WT, intact PMNL, and platelets. *n* = 3–6, (-) not tested

### Inhibition of 15-HETE formation in PMNL and 12-HETE formation in platelets

To investigate the selectivity profile of the inhibitors towards other human lipoxygenases, the formation of 15-HETE, in PMNL, and for selected compounds 12-HETE in platelets was measured (12-LO (gene ALOX12) and 15-LO products (derived from ALOX15 and/or ALOX15B), respectively) (refer to Tables 1, 2, and 3). First, none of the selected compounds tested in platelets was able to affect 12-HETE formation. Second, no substance containing the CCA or BMN scaffold showed an inhibitory effect on 15-HETE formation. Third, within the CAPE-like group, a moderate inhibition of 15-HETE formation was observed. The data indicated an influence of linker length and bulky substituents on 15-LO inhibition. In comparison to CAPE that inhibited 15-HETE production with an IC_50_ of 10 µM, AG556, having a C4 linker, demonstrated an IC_50_ of 5.1 µM. On the other hand, AG494, lacking a linker, did not show a substantial inhibition of 15-HETE formation up to 30 µM. SU1498 did not show inhibition of 15-HETE formation besides containing a C3 linker. However, in contrast to AG556, this compound does not contain a catechol but a single phenolic hydroxyl adjacent to two bulky isopropyl residues. Degrasyn (WP1130) and WP1066 differ from the previous mentioned compounds by lacking phenolic hydroxyl groups but having a 6-bromo-2-pyridinyl moiety instead. In addition, these compounds have a branched alkyl linker. This structural alteration also led to potent 5-LO inhibition with moderate inhibition of 15-HETE formation. In summary, only the CAPE-like inhibitors showed weak inhibition of 15-HETE formation, but the inhibitory effect on 5-LO was at least 10- to 40-fold stronger.

### Effects of cysteine mutations and GSH on 5-LO inhibition of selected compounds

**Fig. 1:**
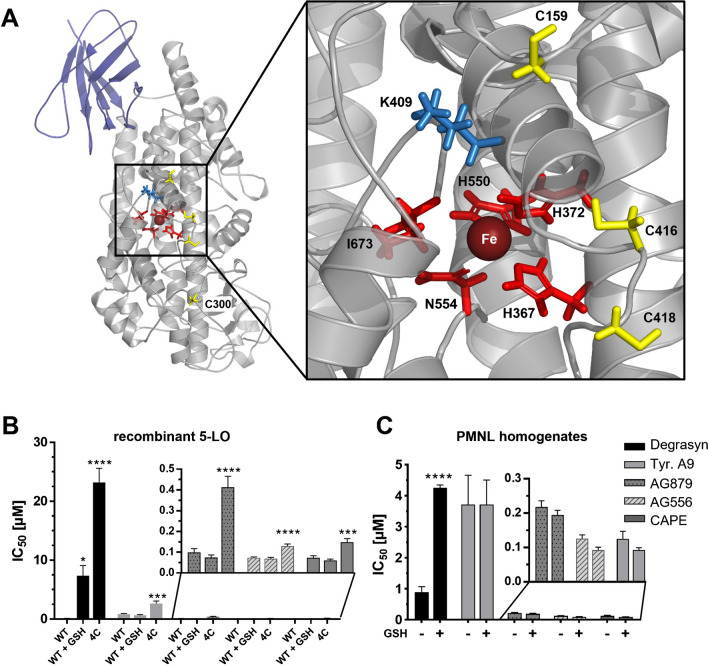
**A** Model of human 5-LO structure based on PDB 3O8Y [17]. 5-LO consists of a C2-like domain (dark blue) and a catalytic domain (grey) which contains the active site with the non-heme iron (dark red) and the iron coordinating amino acids (red). The mutated cysteines C159, C416, and C418 (yellow) are located near the putative entrance to the active site (K409, blue). **B, C** IC_50_ values of selected 5-LO inhibitors in **B** cell-free assays using r5-LO_WT with or without the addition of 1 mM GSH (WT + GSH) or r5-LO_4C, or **C** with sonicated human PMNL with or without the addition of 1 mM GSH. In brief, 3 µg of protein (**B**) or 7.5 × 10^6^ sonicated cells/mL (**C**) were preincubated with an inhibitor in PBS/EDTA/ATP with or without 1 mM GSH for 15 min on ice. The reaction was started by the addition of 2 mM CaCl_2_ and 10 (**B**) or 20 µM (**C**) AA for 10 min at 37 °C. Error bars depict standard deviation of IC_50_. Asterisks indicate significant changes towards r5-LO_WT with the same inhibitor treatment. *(*P* < 0.05), **(*P* < 0.01), ***(*P* < 0.001); ****(*P* < 0.0001), *n* = 3

Previous work revealed that several Michael-reactive inhibitors such as U73122, melleolides, and nitro fatty acids inhibit 5-LO by covalent bond formation with one or more cysteine residues that are in close proximity to the putative substrate entry site close to K409 (Fig. 1A) [2, 9, 23]. The mutant containing four mutations: C159S, C300S, C416S, and C418S, referred to as r5-LO_4C, exhibits comparable activity to the wild type protein, but it is more weakly inhibited by cysteine-targeting compounds such as U73122 or nitro fatty acids [17, 19]. For further investigation, we selected AG556, AG879, tyrphostin A9, and degrasyn due to their potency in inhibiting 5-LO and their diverse structures and determined IC_50_ values using r5-LO_WT with or without the addition of 1 mM reduced GSH or the r5-LO_4C mutant (Fig. 1B). The addition of GSH to the reaction provides information on whether the compounds targeting cysteines specifically attack 5-LO or are generally reactive towards thiols. Interestingly, all four inhibitors showed less inhibition of the r5-LO_4C mutant compared to r5LO-WT. Among the inhibitors tested, degrasyn showed the greatest decrease in potency at r5-LO_4C, with the IC_50_ value increasing more than 200-fold. This effect was less pronounced for tyrphostin A9, AG879, AG556, and CAPE. In contrast, the addition of 1 mM GSH to r5-LO_WT only significantly changed the IC_50_ value for degrasyn, whereas the presence of GSH had no effect on the other compounds.

Next, we evaluated the influence of GSH in PMNL homogenates. Therefore, 7.5 × 10^6^ cells were lysed by sonication, and the assay was performed with or without the addition of 1 mM GSH (Fig. 1C). In agreement with our data measured using recombinant protein, GSH was only able to weaken the inhibitory potency of degrasyn which showed a four-fold increase in the IC_50_ value from 0.89 to 4.2 µM. This finding emphasized the sensitivity of degrasyn reacting non-selectively with thiols and confirmed our previous results using the r5-LO_4C mutant.

**Fig. 2:**
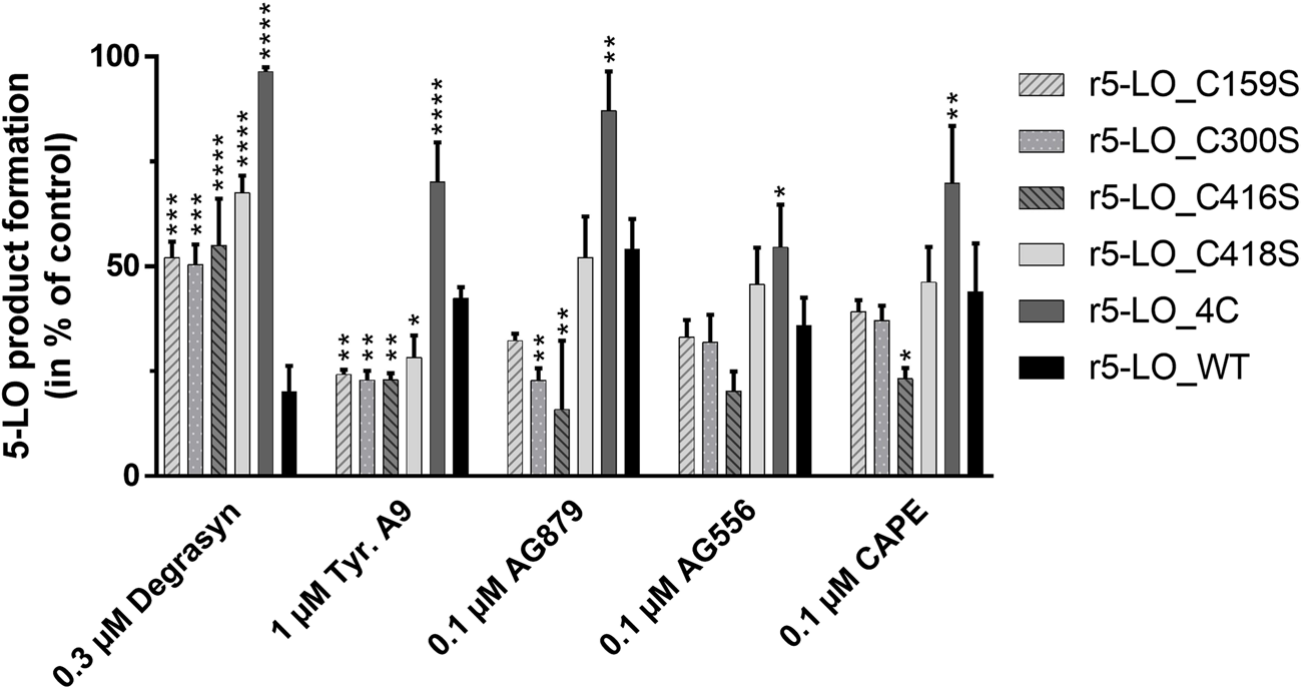
Inhibition of 5-LO single cysteine mutants (C159S, C300S, C416S, or C418S) by degrasyn, tyrphostin A9, AG879, AG556, and CAPE normalized on DMSO control using 3 µg of recombinant 5-LO protein with the respective mutation. The inhibitor concentrations used correspond to the respective IC_50_ value. Inhibitors were preincubated for 15 min in PBS/EDTA/ATP on ice before starting the reaction by the addition of 2 mM CaCl_2_ and 10 µM AA for 10 min at 37 °C. Error bars depict standard deviation. Asterisks indicate significant changes towards r5-LO_WT with the same inhibitor treatment. *(*P* < 0.05), **(*P* < 0.01), ***(*P* < 0.001); ****(*P* < 0.0001), *n* = 3

To ascertain the influence of each individual cysteine mutated in r5-LO_4C, we tested the compounds using the single cysteine mutants C159S, C300S, C416S, and C418S (Fig. 2). While all inhibitors were affected by mutation of all four cysteines, only the effect of degrasyn was significantly impaired by mutation of individual cysteines, irrespective of which of the four cysteines were mutated. Therefore, it seems that all four cysteines appear to be relevant for the inhibition of 5-LO by degrasyn. Surprisingly, for tyrphostin A9, each single mutation enhanced the inhibition of 5-LO. However, the combination of all four led to impaired inhibition of 5-LO. For all inhibitors with the exception of degrasyn, the C416S mutation led to increased 5-LO inhibition.

### Covalent inhibitor binding detected by mass spectrometry

**Fig. 3:**
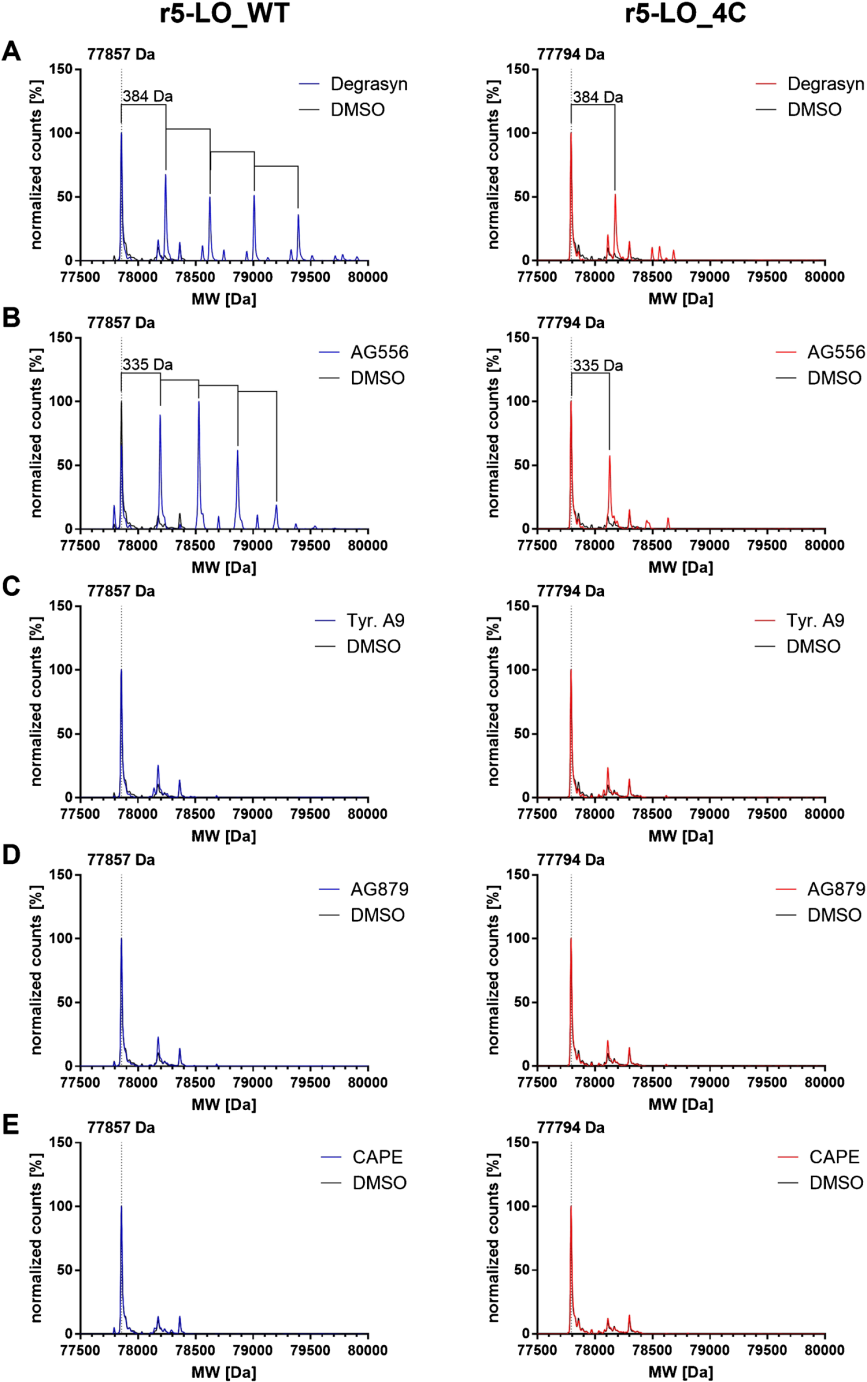
Mass spectra of r5-LO_WT or r5-LO_4C incubated with inhibitors (blue or red line). 20 µM r5-LO_WT (left) or r5-LO_4C (right) was incubated with 100 µM degrasyn (**A**), AG556 (**B**), tyrphostin A9 (**C**), AG879 (**D**), or CAPE (**E**) at RT for at least 1 h before analysis. Measurement was performed using an Agilent 1260 Infinity HPLC with an Agilent 6230 TOF LC/MS detector. Obtained data were processed using Mass Hunter BioConfirm (B.08.00), and resulted counts were normalized to the highest peak of the spectrum

To confirm the covalent binding of the four most potent inhibitors, we incubated r5-LO_WT and r5-LO_4C for 1 h with a fivefold molar excess of the respective inhibitor before subjecting the protein to mass spectrometric analysis, using CAPE as control (Fig. 3). For the unmodified r5-LO_WT and r5-LO_4C, we determined a mass of 77,858 Da and 77,794 Da in agreement with the expected mass based on the primary structure of these proteins. Most interestingly, four peaks corresponding to the expected inhibitor adducts were observed with degrasyn (Fig. 3A) and AG556 (Fig. 3B), corresponding to the mass shifts of 384 Da and 336 Da, respectively. After incubating r5-LO_WT with tyrphostin A9 (Fig. 3C), AG879 (Fig. 3D), and CAPE (Fig. 3E), we did not detect mass shifts. For degrasyn and AG556, a comparison of the MS spectra of r5LO_WT and r5LO_4C revealed the formation of only one adduct with the mutated enzyme. Keeping in mind that 5-LO contains 13 cysteines at least nine of which are exposed at the surface, it is intriguing that three of the cysteines that are located near the entry to the active site appeared to be covalently modified by degrasyn and AG556 (Fig. 1A) [17, 19].

### Cytotoxic effects of the investigated inhibitors

To investigate the cytotoxicity of selected compounds, a multiplex assay was performed using U2OS cells. In this assay, the number of heathy cells were recorded and it simultaneously monitored changes in the phenotypic characteristics of living cells such as changes in nuclear morphology, mitochondrial mass, tubulin structure, and also the effect of inhibitors on membrane permeabilization. Therefore, DNA, mitochondria, and microtubule were stained with different fluorescent dyes in a multiplex assay format. Changes in mitochondrial mass may indicate cytotoxic events and apoptosis [34], while changes in tubulin structure may indicate cytostatic properties of the compounds on the cytoskeleton. Overall, the phenotypic staining provided a comprehensive overview of potential cell-damaging processes and helps to assess the toxicity of compounds [52].

**Fig. 4:**
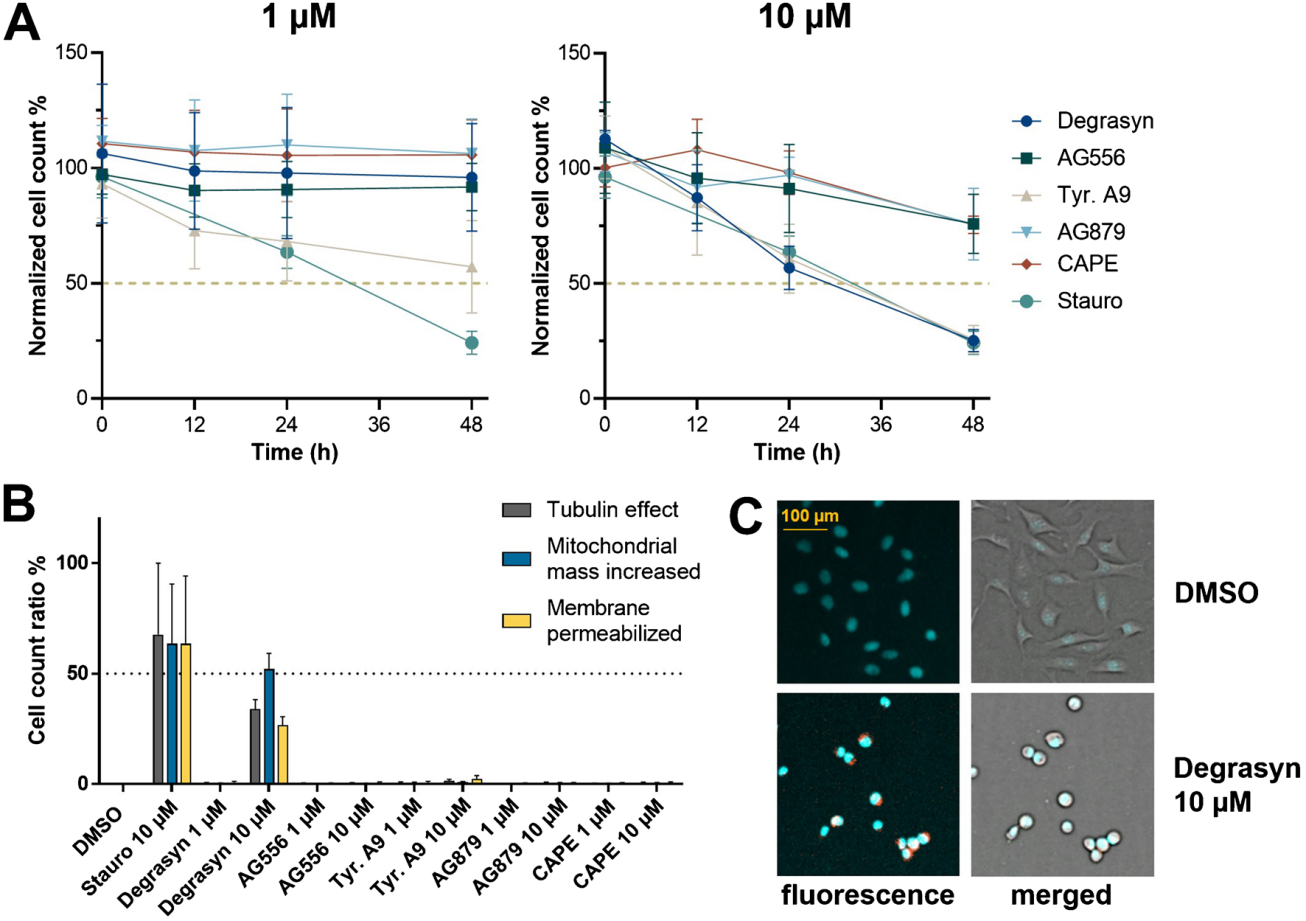
Multiplex assay of U2OS. **A** Normalized healthy cell count after 0 h, 12 h, 24 h, and 48 h after the addition of 1 µM (left panel) or 10 µM (right panel) inhibitor. Staurosporine (10 µM) was used as a control. Live cell count was performed in biological duplicates, normalized to vehicle control (DMSO), and depicted as mean ± SD. **B** Cell count ratio of tubulin effect (grey), increased mitochondrial mass (blue), and permeabilized cellular membrane (yellow) after 48 h. Error bars show SD of biological duplicates. **C** Confocal images of the stained U2OS cells with fluorescent (left panel) and merged brightfield images (right panel) taken after treatment with 10 µM degrasyn for 48 h in comparison to cells exposed to 0.1% DMSO. (Staining: blue: DNA, green: microtubule, red: mitochondria content, magenta: apoptosis marker annexin V)

First, the number of healthy cells was calculated by determining the number of cells at the indicated time points after the addition of 1 µM or 10 µM inhibitor, normalized to the DMSO control (Fig. 4A). Staurosporine, a broad-spectrum protein kinase inhibitor and well-known inducer of apoptosis, served as control and was able to reduce the healthy cell count of U2OS cells to about 25% at a concentration of 10 µM. At 1 µM, none of the tested compounds caused a decrease in healthy cells of more than 50% over a period of 48 h. Only tyrphostin A9 reduced the cell viability about 50%. At 10 µM, tyrphostin A9 and degrasyn reduced the healthy cell count below 50%, whereas the other compounds AG556, AG879, and CAPE reduced the count to around 75%. Overall, only tyrphostin A9 showed clear cytotoxicity at the concentrations required for potent 5-LO inhibition (1 µM).

Next, we observed the phenotypic characteristics of the cells as these can reveal diverse aspects of cytotoxicity beyond the mere survival rate. Figure 4B displays the effects of the selected compounds on tubulin, the increase in mitochondrial mass, and the membrane permeability in U2OS cells. At 1 µM, all compounds were well tolerated and none of them affected the investigated phenotypical properties. At 10 µM, most compounds did not induce any effect; only degrasyn increased the mitochondrial mass by more than 50% and had additional effects on microtubules and on membrane integrity. Furthermore, an assessment of cell morphology showed that at a concentration of 10 µM, degrasyn-treated cells were spherical in shape, indicating cellular stress (Fig. 4C).

## Discussion

### Structure activity relationship

Although there are numerous potential pharmacological applications of LT inhibition by directly targeting 5-LO, this treatment option remains relatively underutilized. For the development of 5-LO inhibiting small molecules, three major types of inhibitors have been used: iron-chelating ligands such as zileuton, redox inhibitors (e.g., NDGA), and non-redox inhibitors (e.g., CJ13,610) [20]. Interestingly, several recent publications demonstrated that 5-LO can be selectively inhibited by Michael-reactive substances such as the known phospholipase C inhibitor U73122, nitro fatty acids, or melleolides from honey mushroom [2, 19, 23]. In this study, we proposed caffeic acid derivatives containing a cyanoacrylate motif as potential lead structures for the further exploration of 5-LO inhibitors with a possible covalent binding mode. We identified the tyrphostins AG879, AG556, and A9 as well as the deubiquitinase and kinase inhibitor degrasyn as potent inhibitors of LT formation that represent a novel structural class of direct 5-LO inhibitors. CCA and CAPE served as template structures; thus, it was not surprising that inhibitors with catechol structures yielded potent 5-LO inhibitors as some known 5-LO inhibitors already contain this motif, e.g., various flavonoids, heteroaryl-substituted catechols, or CAPE [15, 25, 28]. In general, CCA-derived tyrphostins investigated in this study that retain the original carboxylic acid group, such as in AG30, are unable to inhibit 5-LO. However, when this moiety is replaced by the corresponding amide, as present in AG99, strong 5-LO inhibitors were obtained. Therefore, the amide function seemed to be essential for 5-LO potency for this compound class. Comparing the IC_50_ values of AG99 and AG879, it became clear that bulky alkyl substituents at the phenyl ring that form a 3,5-di-*tert*-butylphenyl residue were particularly favorable and reduced the IC_50_ value by a factor of 17. The role of this substitution pattern has already been described for the development of potent 5-LO inhibitors [36]. This principle also applies to tyrphostins 8 and A9, where this substitution converts an inactive compound to a 5-LO inhibitor with an IC_50_ value in the low micromolar range. Adding a third hydroxyl group to the phenyl ring to form a pyrogallol residue in AG82 was also well tolerated with a slight increase of the IC_50_ in intact PMNL compared to the catechol motif. This effect may be partially caused by the increasing polarity of the compound and the resulting loss in cell membrane permeability. Inhibitors with pyridine acetonitrile structure were ineffective in the cell-free assay, but surprisingly, RG14620 was an inhibitor of LT formation in intact PMNL, pointing to a 5-LO-independent inhibition of LT biosynthesis, e.g., by FLAP inhibition. Towards human 12- and 15-HETE formation, the investigated compounds demonstrated selectivity for 5-LO product formation that was pronounced among the CCA and BMN derivatives. Nevertheless, within the group of CAPE-like tyrphostins, an improved inhibition of 15-HETE formation was observed. In particular, the extension of the alkyl linker between amide-N and the benzene ring appeared to lead to reduced selectivity, and this modification increased inhibition of 15-HETE, while the addition of bulky groups on the phenyl ring again led to complete selectivity for 5-LO. Surprisingly, a single mutation from cysteines to serines resulted in good or even better inhibition of 5-LO especially for C416S mutation in all our lead compounds except degrasyn. One could speculate that the C416S mutation leads to a structural change or that the introduction of an H-bridge donor at this site enables better binding of the inhibitors.

### Covalent binding mode

In contrast to the catechol-containing 5-LO inhibitors already described, the structures identified here possessed a cyanoacrylate moiety that enabled covalent binding to cysteines of the enzyme [47]. Our data showed that degrasyn and AG556 were covalently attached to several cysteines, three of which are in close proximity to the putative entrance of the active site [17]. Mutation of these cysteines (C159S, C300S, C416S, and C418S) led to a reduction in the inhibitory effect of all inhibitors; this effect was most pronounced with degrasyn. Nevertheless, mass spec analysis revealed that one covalent attachment of degrasyn or AG556 persisted with the r5-LO_4C mutant. In the case of degrasyn, studies with single mutants showed that none of the cysteines alone was responsible for the inhibitory effect. Besides, the addition of exogenous thiols affected the inhibition by degrasyn on purified protein and in PMNL homogenates, making it possibly sensitive to high intracellular glutathione levels, which may limit its efficacy. For all other compounds, the addition of thiols had no impact on the inhibitory potency. Nonetheless, we were able to show that degrasyn is a potent 5-LO inhibitor in intact PMNL with intracellular GSH present. In contrast, U73122, which had previously been recognized by our group as a covalent, cysteine directed inhibitor, was undesirably ineffective in that setting with an IC_50_ of around 10 µM in intact cells [19]. Besides U73122, nitro fatty acids have been reported to covalently inhibit 5-LO by binding C418 [2]. In addition, the EGFR inhibitor AG556 was also covalently bound to cysteines, but its inhibitory effect was not diminished in presence of GSH and also the mutation of cysteines to serines had only a minor effect. Obviously, the inhibitory mechanisms of degrasyn and AG556 were different. One may question if the thiol reactive property of AG556 was even necessary for its inhibitory potency given that the structurally very similar compound CAPE is also a potent 5-LO inhibitor that is able to inhibit 5-LO as radical scavenger via its catechol structure [6]. Ideally, this covalent ability should result in a prolonged residual time at the target protein with minimal alterations to its structure.

For targets other than 5-LO, Michael acceptors as covalent inhibitors have already been established: In the past decade, we have seen the successful approval of ibrutinib and afatinib in 2013, followed by osimertinib (2015), neratinib (2017), and most recently sotorasib (2021) [1, 5, 10, 16, 37]. All of these compounds share an acrylamide moiety facilitating covalent binding to crucial cysteines via Michael addition making them effective as well as selective tyrosine kinase inhibitors [32]. Unlike simple acrylamides, the addition of a nitrile to the alpha-carbon makes the Michael acceptor more reactive while at the same time the resulting bond becomes reversible [7, 41]. Depending on the substitutional pattern, their reactivity can be adjusted, e.g., strong electron withdrawing groups create more reactive cyanoacrylates [47]. Taking that into consideration, it seems not much of a surprise that degrasyn promiscuously binds thiols. This has also been reported in another context for degrasyn and bacterial proteins [29]. The reaction of AG556 on the other hand is remarkable because the catechol structure is not electron withdrawing towards the acrylonitrile. For tyrphostin A9 and AG879, the fact that we could not detect adducts to r5-LO_WT might be explained by the electron pushing effects or steric shielding of the 3,5-di-*tert*-butylphenol.

In the future, identifying the exact mode of binding may reveal how compounds need to be orientated towards cysteines at the binding site and how they have to be substituted to promote selective covalent binding.

### Multi-target effects

The cytotoxicity assessment revealed only minor impairment of cell viability at 1 µM. Tyrphostin A9 was the only compound that caused a reduction of the normalized cell count to 57%. As tyrphostins were designed for kinase inhibition, e.g., AG879 and tyrphostin A9 inhibit the functions of the growth factor receptor tyrosine kinases HER2/ErbB2 and PDGF, we expected to detect a certain loss of cell viability [30]. Surprisingly, the compounds were overall well-tolerated at concentrations required for effective 5-LO inhibition.

However, not only living cell count was monitored but also the phenotypic properties. The deubiquitinase inhibitor degrasyn caused a noticeable tubulin effect and increased mitochondrial mass [22]. Therefore, our results support the reported proapoptotic properties of the compound [4]. Consistent with this effect, degrasyn can also act as an effective chemosensitizer in various cancer model systems [33, 40, 54].

AG556 acts as an EGFR inhibitor and, although the role of 5-LO in tumor development and progression is not yet fully understood, combined EGFR and 5-LO inhibition was found to be beneficial [21, 42]. In such cases, dual inhibitors of tyrosine kinases and 5-LO may be of interest.

## Conclusion

Our results allow us to propose novel lead structures for potent and covalent 5-LO inhibitors. We found that the tyrphostins A9, AG556, AG879, and the deubiquitinase inhibitor degrasyn effectively suppressed LT production in cellular assay systems and proved to be selective for 5-LO over 15-LO and platelet 12-LO. Additionally, we were able to identify a covalent binding mode for degrasyn and AG556. The multiplex assay results reflected the proapoptotic properties of degrasyn, as it caused phenotypic changes of the cells. Nevertheless, these effects were only observed at concentrations above those required for 5-LO inhibition. Ultimately, these multi-target effects of the investigated compounds have to be taken into account for further development as 5-LO inhibitors.

In conclusion, our findings introduce a novel class of 5-LO inhibitors with possible covalent binding mode that exhibits high potency and highlights possibilities of covalent inhibitor development for this undervalued pharmacological target.

## Data Availability

Data will be made available on request.
